# Individuals’ Decision to Co-Donate or Donate Alone: An Archival Study of Married Whole Body Donors in Hawaii

**DOI:** 10.1371/journal.pone.0042673

**Published:** 2012-08-07

**Authors:** Michel Anteby, Filiz Garip, Paul V. Martorana, Scott Lozanoff

**Affiliations:** 1 Organizational Behavior Area, Harvard Business School, Boston, Massachusetts, United States of America; 2 Department of Sociology, Harvard University, Cambridge, Massachusetts, United States of America; 3 Business Administration Department, Wagner College, New York, New York, United States of America; 4 Department of Anatomy, Biochemistry and Physiology, University of Hawaii, Honolulu, Hawaii, United States of America; University of Illinois at Champaign-Urbana, United States of America

## Abstract

**Background:**

Human cadavers are crucial to numerous aspects of health care, including initial and continuing training of medical doctors and advancement of medical research. Concerns have periodically been raised about the limited number of whole body donations. Little is known, however, about a unique form of donation, namely co-donations or instances when married individuals decide to register at the same time as their spouse as whole body donors. Our study aims to determine the extent of whole body co-donation and individual factors that might influence co-donation.

**Methods and Findings:**

We reviewed all records of registrants to the University of Hawaii Medical School’s whole body donation program from 1967 through 2006 to identify married registrants. We then examined the 806 married individuals’ characteristics to understand their decision to register alone or with their spouse. We found that married individuals who registered at the same time as their spouse accounted for 38.2 percent of married registrants. Sex differences provided an initial lens to understand co-donation. Wives were more likely to co-donate than to register alone (p = 0.002). Moreover, registrants’ main occupational background had a significant effect on co-donations (p = 0.001). Married registrants (regardless of sex) in female-gendered occupations were more likely to co-donate than to donate alone (p = 0.014). Female-gendered occupations were defined as ones in which women represented more than 55 percent of the workforce (e.g., preschool teachers). Thus, variations in donors’ occupational backgrounds explained co-donation above and beyond sex differences.

**Conclusions:**

Efforts to secure whole body donations have historically focused on individual donations regardless of donors’ marital status. More attention needs to be paid, however, to co-donations since they represent a non-trivial number of total donations. Also, targeted outreach efforts to male and female members of female-gendered occupations might prove a successful way to increase donations through co-donations.

## Introduction

Human cadavers are crucial to numerous aspects of health care, including initial and continuing training of medical doctors and advancement of medical research. Yet medical professionals have repeatedly voiced concerns over a sufficient supply of cadavers. [1], [2], [3] Average estimates of the cadaver supply in the United States put the total at less than 18,000 donations per year. [4] Recent developments in surgical procedures requiring cadaveric testing and training have only heightened the concerns.

While a few states still rely on unclaimed cadavers to help meet their needs, uncompensated voluntary whole body donations are overwhelmingly favored in the United States. The1968 Uniform Anatomical Gift Act provided an initial legal framework for such donations. [5] The Act’s 1987 revision made it a felony to “knowingly, for valuable consideration, purchase or sell a [body] part for transplantation or therapy, if removal of the part is intended to occur after the death of the decedent” and a later revision confirmed this view. [6], [7] Though prohibitions on the purchase and sale of whole bodies, as opposed to body parts, and for purposes other than transplant or therapy (particular, education and research) are not specifically addressed by the Act, its scope is usually seen as also encompassing whole body donations regardless of purpose. Thus, a better understanding of donors’ behavior is important to securing cadavers.

Most past research on cadaveric donations has focused on individual donations as focal units of analysis and often emphasized sex differences in explaining such donations. [8], [9] Indeed, in the context of voluntary donations, individuals are generally viewed as key decision-makers. Because altruistic behaviors are known to differ by gender, sex differences prove an important lens through which to examine donors’ behaviors. [10] Also, men and women’s contrasted attitudes toward cadaveric donation support such an approach. [3], [11] Even when findings are embedded in larger community dynamics, individual donations remain the default unit of analysis and sex differences an important factor in explaining donation.

Yet donors rarely act alone and sometimes even donate at the same time as others. Past studies of non-cadaveric donation suggest that donating decisions often include close family members or friends. [12], [13], [14] Also, a number of registered cadaveric donors report knowing someone intending to donate. [15] One past study of cadaveric donors even notes, without exploring underlying reasons, a tendency for married couples to donate together. [8] Despite this evidence, the phenomenon of married co-donation or instances when married individuals decide to register at the same time as their spouse as whole body donors has remained largely unexplored. The goal of this study is to examine the extent of co-donation among whole body donor registrants and to explore individual determinants of married registrants’ decision to co-donate.

## Methods

### Study Setting

We intentionally selected the University of Hawaii’s whole body donation program to minimize the risk of not capturing all registrations occurring in the selected geography. The University of Hawaii was the sole procurer of whole body donations in the state of Hawaii during the period of study; the likelihood of Hawaii whole body donors not registering with the program was extremely low. Registration by Hawaii residents with out-of-state programs while theoretically possible was logistically highly unlikely due to Hawaii’s remote geographical location. Thus, our data likely encompass not only a sample of registrants but the entire population of whole body donation registrants in Hawaii.

### Case Accrual Procedure

We relied on 1,746 archived records of registered donors to the University of Hawaii’s whole body donation program from 1967 to 2006 to identify a subset of that population that could engage in co-donation, namely, married registrants. The program operated on a model typical of most other U.S. donation programs. [16] The records constitute the entire sample of complete registrations received by the program from its inception in 1967 to 2006, the most recent year for which data were available at the time of collection.

Registration as a cadaveric donor captures individuals’ behavior better than ultimate cadaveric donation. In Hawaii, as in most U.S. states, the deceased need not have explicitly agreed to donation; parents and close relatives, for instance, can donate the body of their deceased kin. [17] Hawaii legislation governing donation states that the persons otherwise entitled by law to control the disposition of a decedent’s body shall faithfully carry out the directions of the decedent, but no proof of such directions is required. [18] In that sense, the act of registering to donate one’s body is a strong proxy for donating, since it alone clearly articulates the intent to donate.

### Data Collection Procedure

Potential donors who had requested information (usually by phone or mail) returned signed forms indicating their intent to donate their bodies after death. Each donor required a separate registration form, and only one registration form was mailed to each requestor unless the requestor asked for more than one. Records include each registrant’s full name, year of birth, date of registration, sex, race/ethnicity, religious affiliation, marital status, and primary (current or past) occupation. Married registrants were asked to provide their spouses’ names. All registrants were also asked to disclose the names of their father, mother, legal next of kin, and children.

### Ethics Statement

Access to the data was granted by the program director after the University of Hawaii and Harvard University institutional review boards’ approvals. The institutional review boards waived the need for written informed consent from participants in light of Title 45, part 46, section 116 (d) in the U.S. Code of Federal Regulations since: (a) no more than minimal risk to the subjects was identified; (b) a waiver or alteration did not adversely affect the rights and welfare of the subjects; (c) the study could not practicably be carried out without the waiver or alteration; and (d) whenever appropriate, the subjects would be provided with additional pertinent information after participation. Confidentiality was ensured by deleting registrants’ names following coding of donor relationships.

### Variables

Our dependent variable captured whether married individuals registered alone or at the same time as their spouse. All known relationships between registrants were coded to identify instances of co-donations. Spousal relationships were the most common; other relationships, such as father–son and brother–sister, were so rare (n = 15 of 1,746 total registrants) that we decided to limit our attention to spousal co-donations. Spouses were coded as married co-registrants (coded as 1) if they (a) were married and (b) had both registered the same day as whole body donors. In 78.2% of the cases, the husbands and wives registered on the same day. Spouses both registering as donors but on different days were not considered married co-registrants. Married co-registrants were identified by searching the registry for the spouse listed by each focal registrant. If a matching name was found, and if both spouses registered the same day, both were coded as married co-registrants. Individuals were coded as married solo registrants (coded as 0) if they were (a) married and (b) had registered alone as whole body donors to the University of Hawaii program.

Key independent variables: Sex was coded 1 for female and 0 for male. Registrants listed their main occupation prior to retirement as the type of job they had engaged in or were engaged in for most of their working lives at the time of registration. Each registrant’s occupation was matched with the percentage of women working in that occupation in all U.S. states between 1983 and 1987, roughly the mid-point of our study period. [19] Female-gendered occupation was coded 1 if females made up more than 55 percent of the workforce in that occupation and 0 if not (see Table 1 for top female-gendered occupations). We used a cut-off point because an occupation is hypothesized to be female-gendered only after it reaches a threshold of female representation. [20] Respondents who listed homemaker as an occupation were coded as working in a female-gendered occupation. Registrants whose reported occupations were not immediately categorizable (11.4% of all registrants) were categorized independently by two coders into the closest matching occupational categories used by the U.S. Department of Labor; coding of these cases yielded a 98-percent inter-rater reliability.

**Table 1:** Top 10 Female-Gendered Occupations among Registrants*.

Occupation	Percentage Female
Executive Secretaries and Administrative Assistants	99.1
Secretaries, Except Legal, Medical, and Executive	99.1
Preschool Teachers, Except Special Education	98.4
Dental Assistants	98
Receptionists and Information Clerks	97.5
Child Care Workers	96.7
Food Preparation Workers	96.3
Personal and Home Care Aides	96
Education, Training, and Library Workers, All Other	95.1
Licensed Practical and Licensed Vocational Nurses	95.1

* Percentage female based on the 1987 Current Population Survey. Homemakers not included.

Socio-demographic controls: Registrants’ self-reported Race/Ethnicity was coded in keeping with the major U.S. Census categories; some categories were then consolidated to reflect unique data. Caucasian (n = 1,266) and Asian (n = 340) registrants were most heavily represented and were therefore assigned independent categories. The few registrants reporting other races/ethnicities, namely Native Hawaiian and Other Pacific Islander (n = 8), Black/African American (n = 11), Hispanic/Latino (n = 9) and more than one race (n = 112), were consolidated into a single “other race/ethnicity” category. To account for possible age variations, we included age at registration as a control variable. In keeping with U.S. Census main categories, religious affiliation was initially coded as “Christian,” “other religious affiliation” or “secular.” Given the centrality of faith with respect to disposition of the dead, we subsequently collapsed Christians and other religious affiliations into a single category labeled “observant” (coded as 0) or “secular” (coded as 1). To account for possible historical variations in registration patterns, we included dummy variables (n = 39) for each year of registration in the study (1967–2006). Each registrant’s occupation was also matched with the estimated mean annual wage for that occupation in Hawaii. [21] Homemakers were attributed an estimated annual wage of zero. Annual wages were used in logarithm form to account for the skewness in distribution.

### Statistical Analysis

Our analyses proceeded in two stages. We first focused on all married registrants to understand why some married registrants opted to co-register while others registered alone (Table 2). Because sex differences are commonly analyzed with respect to cadaveric donations, we then investigated married men and women separately. In our analysis of all married registrants, we estimated a series of binary logistic regression models measuring the effects of sex and working in a female-gendered occupation on co-registration. The models are organized hierarchically in Table 3. Model 1 examines the association between sex and co-registration. Model 2 examines the association of working in a female-gendered occupation and co-registration. Model 3 includes both sex and the indicator for female-gendered occupation to test their effects on co-registration.

**Table 2:** Registrant Demographics.

Variables	All MarriedRegistrants *N* = 806	Married SoloRegistrants *n* = 498	Married Co-Registrants*n* = 308	p
Female	361 (44.8)	207 (41.6)	154 (50.0)	0.00017
Female-gendered occupation	323 (40.1)	176 (35.3)	147 (47.7)	0.00049
Age at registration	67.9 (12.9)	66.8 (13.1)	69.6 (12.4)	0.00261
Race/Ethnicity				
Asian	174 (21.6)	111 (22.3)	63 (20.5)	0.45718
Caucasian	573 (71.1)	347 (69.7)	226 (73.4)	
Other	59 (7.3)	40 (8.0)	19 (6.2)	
Religious Affiliation				
Observant	362 (44.9)	223 (44.8)	139 (45.1)	0.97902
Christian	314 (39.0)	194 (39.0)	120 (39.0)	
Other	48 (6.0)	29 (5.8)	19 (6.2)	
Secular	444 (55.1)	275 (55.2)	169 (54.9)	
Estimated annual wages (in U.S. dollars)	50617	52139	48156	0.06345

** p<.01, *p<.05, ^†^p<0.10 (two-tailed). Female-gendered occupations contain more than 55% women. Figures in parentheses are percentages except for age and estimated annual wages, in which case they are standard deviations. The last column shows t-test (two-tailed) and chi-squared p-values to compare married solo registrants to married co-registrants. For the female indicator, the chi-squared value (14.17) is computed by setting the expected cell count for male solo donors equal to that for female solo donors (498/2 = 249), which is the expected outcome under the null hypothesis of independence between sex and co-donation status. (We thank an anonymous reviewer for pointing this out.).

**Table 3:** Logistic Regression Models of Sex and Occupation on Co-Registration.

Variables	Model 1	p	Model 2	p	Model 3	p
Female	1.44	0.002	–		1.09	0.599
	(0.17)				(0.18)	
Female-gendered occupation	–		1.76	0.001	1.67	0.014
			(0.28)		(0.35)	
Age at registration	1.02	0.003	1.02	0.004	1.02	0.004
	(0.01)		(0.01)		(0.01)	
Asian	1.00	0.996	0.98	0.924	0.97	0.906
	(0.23)		(0.23)		(0.23)	
Other race/ethnicity	0.95	0.863	0.91	0.772	0.91	0.777
	(0.30)		(0.30)		(0.30)	
Secular	0.92	0.679	0.90	0.586	0.90	0.612
	(0.19)		(0.18)		(0.18)	
Log of estimated annual wages	0.97	0.343	0.99	0.725	0.99	0.782
	(0.03)		(0.03)		(0.03)	
Year dummies	yes		yes		Yes	
N	806		806		806	
Pseudo-R^2^	0.07		0.08		0.08	
Likelihood ratio test (models 1 & 3)					5.98	0.015
Likelihood ratio test (models 2 & 3)					0.17	0.678

Results presented in odds ratios. Standard errors, shown in parentheses, are adjusted for couple-level clustering. Female-gendered occupations contain more than 55% women. Caucasian is the reference category for race/ethnicity. Observant is the reference category for religion.

To determine whether the effect of working in a female-gendered occupation on co-registration differed by sex, we re-calculated our regression in Model 3 for men and women separately in Table 4. All models include controls for key socio-demographic categories. We also adjusted all our model estimates for couple-level clustering. No variables were multicollinear in any of our models (variance inflation factors <2) [22].

**Table 4:** Logistic Regression Models of Occupation on Co-Registration by Sex.

Variables	Men	p	Women	p
Female-gendered occupation	1.92	0.038	1.64	0.082
	(0.61)		(0.47)	
Age at registration	1.02	0.080	1.03	0.001
	(0.01)		(0.01)	
Year dummies	yes		yes	
N	445		361	
Pseudo-R^2^	0.08		0.08	

Results presented in odds ratios. Standard errors, shown in parentheses, are adjusted for couple-level clustering. Female-gendered occupations contain more than 55% women.

## Results

### Study Sample

The program received a total of 810 registrations from married individuals during the period of study. Married registrants accounted for 46.8 percent of the total donor population and constituted the largest of the four marital categories. Divorced, widowed, and never-married registrants respectively represented 22.9 percent, 16.8 percent, and 13.4 percent of all registrants. We excluded two co-registering same-sex couples because they might bias a sex effect. These exclusions gave us a final tally of 806 married registrants from which to examine decisions to engage in co-donation.

Table 2 reports registrants’ demographics. Within our sample, males were more highly represented than females (55.2% and 44.8% respectively). The mean age at registration for male registrants was significantly higher than that of female registrants: 69.7 years versus 65.6 (*p* = 0.00001). Only 13 percent of male registrants worked in female-gendered occupations compared to 73.4 percent of female registrants (*p*<0.00001). The sample was predominantly Caucasian (71.1%). Asians were the second largest category (21.6%). With respect to religious affiliation, a slight majority, 55.1 percent, was secular. Christianity was the predominant religious affiliation, cited by 39 percent of all married registrants. The mean estimated annual wage of the entire population was $50,617 (*S.D.* = $29,605).

Of the 806 married registrants in our sample, co-registrants accounted for 308 or 38.2 percent of the sample. Thus, a significant percentage of all married registrants decided to donate at the same time as their spouses. Co-registrants included a higher percentage of females compared to those who registered solo (50% versus 41.6%, *p* = 0.00017) and a higher percentage of individuals in female-gendered occupations (47.7% versus 35.3%, *p* = 0.001) again compared to those who registered solo. Co-registrants were older than solo-registrants on average (69.6 versus 66.8 years, *p* = 0.003), but earned significantly less ($48,156 versus $52,139, *p* = 0.063).

### Factors Associated with Co-Registration

Results of our hierarchically nested regression analyses show an association of both sex and working in a female-gendered occupation on co-registration by married registrants. First, as Model 1 in Table 3 indicates, sex significantly predicted spousal co-registration (*odds ratio* = 1.44; *p* = 0.002). Husbands were significantly more likely to register alone, while wives were significantly more likely to co-register with their husbands. These results support an interpretation of co-donation based on sex differences. The socioeconomic controls, including the year each individual registered, logarithm of annual estimated wages, race/ethnicity (Caucasian, Asian or Other), and religious affiliation (observant or secular) did not significantly predict spousal co-registration in any of the models. Age at time of registration increased the odds of co-registration in all three models.

As shown in Model 2, working in a female-gendered occupation also influenced spousal co-registration (odds ratio = 1.76; *p* = 0.001). Married registrants in female-dominated occupations were significantly more likely to co-register than their counterparts in other occupations. When both indicators for sex and working in a female-gendered occupation were included in Model 3, the positive effect of sex on co-registration was significantly reduced (*odds ratio* = 1.09, *p* = 0.599). Husbands were less likely to co-register than wives because they were less likely to work in female-gendered occupations. Working in a female-gendered occupation explains spousal co-registration even after controlling for sex differences. This conclusion is confirmed by likelihood-ratio tests, which show that Model 3 provides a significantly better fit compared to Model 1 (*p* = 0.015) but not compared to Model 2 (*p* = 0.678).

Table 4 presents separate models for married men and women to test whether the effect of working in a female-gendered occupation on co-registration differs by sex. The socio-demographic characteristics (race/ethnicity, religion and wages), which were consistently insignificant in Table 3, were excluded to preserve statistical power with the reduced sample sizes. Working in a female-gendered occupation significantly increased the odds of spousal co-registration for both married men (*odds ratio* = 1.92, *p* = 0.038) and married women (*odds ratio* = 1.64, *p* = 0.082). Thus, working in a female-gendered occupation explained co-donation above and beyond sex differences.

In supplementary analyses, we used the alternative cut-off point of 60 percent (instead of 55 percent) to define a female-gendered occupation. The results in the main models of Table 3 remained unchanged. The results in the female-gendered work models of Table 4 were similar in sign and magnitude but not significant, possibly due to the reduced sample sizes. We conducted a power analysis and found that our sample sizes for men (445) and women (361) were only sufficient to detect an effect of working in a female-gendered occupation with the 55 percent cut-off point.

## Discussion

No single solution can increase the number of whole body donors for medical education and research. This study first identifies a previously overlooked form of donation, namely co-donations by married individuals. This study also points to individual donors’ main occupational background, particularly whether they worked in female-gendered occupations, as a key factor influencing co-donation. If a registrant–regardless of sex–had been engaged in a female-gendered occupation, he or she was more likely to co-register. For husbands, in particular, having been engaged in a female-gendered occupation facilitated co-donation.

These results suggest that past views of donors might have overlooked the potential for co-donations. The extent of co-donation (here, 38.2 percent of married registrants) seems consistent with a prior report in another U.S. whole body donation program (32.1 percent). [3] Handling married potential donors’ inquiries might therefore benefit from some revision. Whole body program staff members could, for instance, discuss with such donors the option to co-donate. Further research could also explore non-spousal co-donations (e.g., parent/child) and factors associated with them.

Our methodological choices might have led us to underestimate the extent of co-donations. Co-donations are an example of possible joint behaviors or instances when two or more persons engage in acts judged common or concerted, on one or more dimensions, such as direction, tempo, or substantive content. Our proxy for judging acts to be joint was temporal simultaneity. Yet temporal simultaneity is not the only proxy for joint registration. Spouses deciding on the same day to register as donors might subsequently sign their registrations on different days, for instance, leading to lagged registration dates. (Our results remained robust with lagged registration dates.) The extent to which we coded behaviors as co-donation is therefore probably conservative; co-donation might be more widespread than reported here.

Our study’s findings also point to specific professional circumstances that might be associated with co-donations: implying that whole body donation programs’ outreach efforts might benefit from better targeting. In the same way that charitable giving in the United States is currently sometimes workplace-based, co-donor registration could be encouraged along occupational lines. [23] In particular, targeting members of female-gendered occupational groups–e.g., through their employers or union representatives–might prove useful in securing co-donations. Indeed, securing, for example, co-donations from married pre-school teachers (i.e., a female-gendered occupational group) might prove easier than securing co-donations from married firefighters (i.e., a non female-gendered occupational group).

Several limitations of our study should be acknowledged. First, while our focus on female-gendered occupations provides insight into co-donations, it does not fully capture the complexity of donating decisions. In particular, we do not capture spousal dynamics involved in donating decisions. Also, many other factors, including institutional, have been shown to influence other donation types (e.g., blood or organs). [23], [24] Although we controlled for several key factors that might explain variation in co-donation patterns, we cannot rule out that the observed relationship between female-gendered occupations and co-registration might be caused by an unobserved variable.

Second, our study focused on a setting (Hawaii) with unique geographic and demographic features. As a state made up entirely of islands, remote from the mainland, Hawaii residents might be more inclined than others to make decisions regarding death in light of their spouse’s decision. For married Hawaii residents born on the mainland, final body disposition in their region of birth is probably more complicated to execute than for residents of other U.S. states. Those individuals might be more inclined to make end of life choices with their spouse than they would if they resided on the mainland. Also, Hawaii’s population of potential whole body donors might differ from those of other states, particularly along ethnic lines. Such dimensions might prove important in explaining co-donation in other settings.

### Conclusion

Public overall reluctance toward cadaveric donations and the limited cadaveric supply are matters of repeated concern for the medical profession [2], [3], [11]. Our study suggests novel approaches to securing donations by focusing not only on individual donations, but also on co-donations by married individuals, particularly men and women employed in female-gendered occupations. Though our data are confined to whole body co-donation, these suggestions may apply more broadly to other forms of co-donation. Our findings highlight the fact that occupational groups provide important arenas of socialization that can inform donor behaviors and even explain an individual’s increased odds of co-donating.
